# Macroscopic Portal Vein Thrombosis in HCC Patients

**DOI:** 10.1155/2018/3120185

**Published:** 2018-06-13

**Authors:** Hikmet Akkiz, Brian I. Carr, Sedef Kuran, Ümit Karaoğullarından, Oguz Üsküdar, Salih Tokmak, Burcu Arslan, Figen Doran, Hüseyin Tugsan Balli, Abdulalh Ülkü, Tolga Atılgan Akçam, Halil İbrahim Bahçeci, Kamil Yalçın Polat, Necati Örmeci, Halis Şimşek, Abdullah Sonsuz, Ali Demir, Engin Altıntaş, Mehmet Demir, Kendal Yalçın, Nazım Ekinci, Ayşegül Harmancı Özakyol, Mehmet Yücesoy, Ahmet Uygun, Vito Guerra, Anıl Delik, Yaman Tokat, Sezai Yilmaz, Ahmet Bektaş, Murat Kılıç

**Affiliations:** ^1^Çukurova University Gastroenterology Department, Adana, Turkey; ^2^İzmir Biomedicine and Genome Institute, Dokuz Eylül University, İzmir, Turkey; ^3^Fırat University, Turkey; ^4^İstanbul Memorial Hospital, Turkey; ^5^Ankara University, Turkey; ^6^Hacettepe University, Turkey; ^7^İstanbul Cerrahpaşa University, Turkey; ^8^Konya Necmettin Erbakan University, Turkey; ^9^Mersin University, Turkey; ^10^Hatay Mustafa Kemal University, Turkey; ^11^Dicle University, Turkey; ^12^Eskişehir Osmangazi University, Turkey; ^13^Erciyes University, Turkey; ^14^Haydarpaşa Sultan Abdülhamid Eğitim Araştırma Hospital, Turkey; ^15^Trials Centre, National Institute for Digestive Diseases, IRCCS “Saverio de Bellis”, Castellana, Bari, Italy; ^16^Florence Nightingale Hospital, İstanbul, Turkey; ^17^İnonu University, Malatya, Turkey; ^18^Ondokuz Mayıs University, Turkey; ^19^Izmir Kent Hospital, Turkey

## Abstract

Macroscopic portal vein invasion (PVT) by hepatocellular carcinoma (HCC) in the liver is one of the most important negative prognostic factors for HCC patients. The characteristics of a large cohort of such patients were examined. We found that the percent of patients with PVT significantly increased with increasing maximum tumor diameter (MTD), from 13.7% with tumors of MTD <5cm to 56.4% with tumors of MTD >10cm. There were similar numbers of HCC patients with very large tumors with and without PVT. Thus, MTD alone was insufficient to explain the presence of PVT, as were high AFP levels, since less than 50% of high AFP patients had PVT. However, the percent of patients with PVT was also found to significantly increase with increasing blood alpha-fetoprotein (AFP) levels and tumor multifocality. A logistic regression model that included these 3 factors together showed an odds ratio of 17.9 for the combination of MTD>5.0cm plus tumor multifocality plus elevated AFP, compared to low levels of these 3 parameters. The presence or absence of macroscopic PVT may therefore represent different HCC aggressiveness phenotypes, as judged by a significant increase in tumor multifocality and AFP levels in the PVT positive patients. Factors in addition to MTD and AFP must also contribute to PVT development.

## 1. Introduction

The prognosis of patients with hepatocellular carcinoma (HCC) depends upon both tumor factors and liver factors [1]. The tumor factors include the maximum tumor diameter (MTD), number of tumors, presence of macroscopic (clinically evident) portal vein thrombosis (PVT), and blood levels of alpha-fetoprotein (AFP). The presence of PVT may be the most important tumor factor, as it reflects tumor aggressiveness (migration, invasion, and potential for metastasis), limits the options for curative resection or transplantation, and can also worsen residual liver function. It is thought that up to 45% of HCC patients have some form of macroscopic PVT [2–5] and may be gross or macroscopic as shown on CT or MRI scan or microscopic as evidenced only on pathology. It is poorly understood, with few biological models and little understanding of its causes. However, predisposing factors include increased MTD, with increased levels of the HCC plasma tumor markers des-gamma carboxyprothrombin (DCP) and AFP, decreased serum albumin, and elevated platelet counts [6–11]. The current study in a large HCC cohort confirms the increased percent of PVT with increasing MTD, as well as with increasing multifocality and AFP. Furthermore, we found that PVT occurred in 13.7% of small HCCs <5cm, but in 56.4% of large HCCs >10cm.

## 2. Methods

### 2.1. Patient Data

We analyzed a database of 1773 prospectively accrued HCC patients who had full baseline tumor parameter data, including CT scan information on HCC size, number of tumor nodules, and presence or absence of PVT and plasma AFP levels; complete blood count; routine blood liver function tests (total bilirubin, GGTP, ALKP, albumin, and transaminases); and patient demographics. Diagnosis was made either via tumor biopsy or according to international guidelines. Of these patients, 1029 had low AFP levels (≤100 IU/ml) and are the subject of this study. Database management conformed to legislation on privacy and this study conforms to the ethical guidelines of the Declaration of Helsinki and approval for this retrospective study on deidentified HCC patients was obtained by the Institutional Review Board.

### 2.2. Statistical Analysis

Mean and SD for continuous variables and relative frequency for categorical variables were used as indices of centrality and dispersion of the distribution. For categorical variables, the Chi-square and z test for proportions were used. The Wilcoxon rank-sum (Mann–Whitney) test was to test the difference between two categories and the Kruskal-Wallis rank test to test the difference among categories.

Logistic regression model was to evaluate the associations between PVT (No/Yes) on single variables examined.

Final multiple linear or logistic regression models were obtained with the backward stepwise method and the variables that showed associations with p<0.10 were left in the models.

When testing the null hypothesis of no association, the probability level of *α* error, two tailed, was 0.05. All the statistical computations were made using STATA 12.1 Statistical Software (StataCorp), 2014, release 12 (College Station, TX).

## 3. Results

### 3.1. PVT in Tumor Size (MTD) Groups

Patients were initially analyzed according to PVT status in different tumor size (MTD) groups (Table 1). There was a large percent increase in PVT positive patients with increase in MTD, 13.7% for small tumors, 33.9% for intermediate tumors, and 56.4% for very large tumors. For small and intermediate size tumor patients, there were more Child-Pugh score A patients without PVT. However, the prevalence of cirrhosis was not different across MTD groups, nor were there differences in total serum bilirubin values. Albumin values were lower in the patients with PVT, but significant only for the small tumor groups. Patients with PVT had a small but significant increase in MTD, in the small and intermediate size tumor groups compared to patients without PVT. However, the AFP levels were significantly higher in the PVT positive patients, for all 3 tumor size groups, as was tumor multifocality. Thus, on the whole, patients with PVT had larger and more multifocal tumors with higher AFP values, yet similar bilirubin levels, across the tumor size groups.

### 3.2. PVT-Associated Parameters

**Figure 1:**
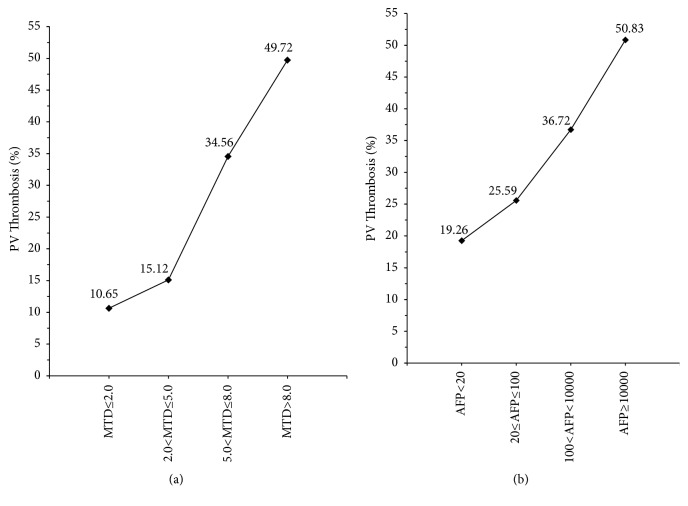
Percentage of patients with PV Thrombosis in** (a) **MTD (cm) categories, in total cohort (p <0.0001^*∗*^)^;^** (b) **AFP (IU/mL) categories (p <0.0001^*∗*^)^;^*∗* Chi-square test for trend; PVT, portal vein thrombosis; MTD, maximum tumor dimension; AFP, alpha-fetoprotein.

**Figure 2:**
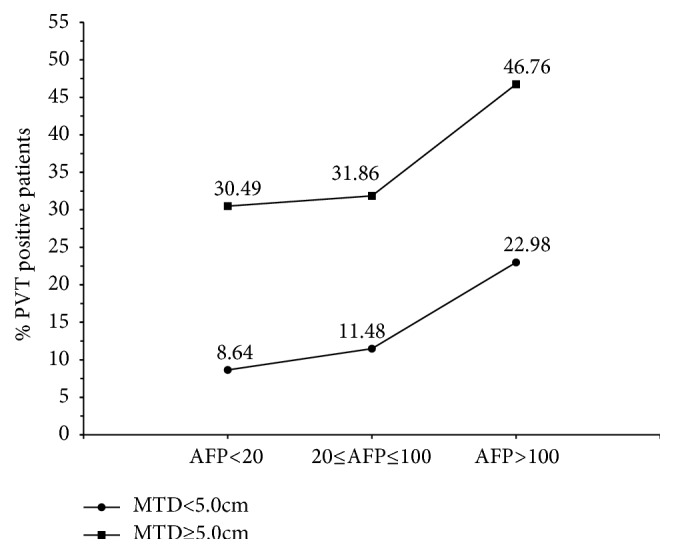
Percentage of patients with PVT in MTD (cm) categories: MTD<5.0cm (p <0.0001^*∗*^) and MTD≥5.0cm (p <0.0001^*∗*^)^.^ ^*∗*^ Chi-square test for trend; PVT, portal vein thrombosis; MTD, maximum tumor dimension (cm); AFP, alpha-fetoprotein (IU/mL).

The parameters that were associated with presence of PVT were next examined. A logistic regression model of PVT showed significance for several single parameters as continuous variables (Table 2(A)), but when all parameters were considered together, there was significance in the final model (Table 2(B)) for tumor multifocality, MTD, AFP, ALKP, and albumin. Two of these parameters versus PVT values are shown graphically in Figure 1, which shows significant increases in percent PVT, in relation to increase of both MTD and AFP. However, even in the highest AFP categories, less than r50% of patients had PVT (Figure 2).

A logistic regression model of PVT was then performed for the variables of MTD (large or small), AFP (high or low), and tumor multifocality versus unifocality, considered singularly (Table 3(A)) or together (Table 3(B)), as these were the 3 parameters with highest odds ratio (OR) in Table 2. High ORs were found for the high values of each of the 3 parameters, whether considered singularly or together. However, when we combined all 3 parameters of MTD >5cm plus tumor multifocality plus AFP >100 IU/ml, an OR of 17.9 was obtained (Table 3(C)).

## 4. Discussion

Clinical macroscopic PVT is typically diagnosed as obstruction and expansion of the portal vein on CT or MRI scan [12–14] or by contrast-enhanced ultrasonography [15]. It is associated with several serum changes, such as the des-gamma-carboxy-prothrombin/vitamin K pathway [16–23] and is a well-recognized predictor of poor survival in HCC patients [1, 5–7, 24–26]. It is typically diagnosed radiologically [13–15] and although pathological confirmation often requires examination of resected or transplantation specimens, percutaneous biopsy material can also be used [27, 28]. Despite this, some factors have been associated with discriminate better survival among patients with PVT. These include serum albumin levels [9], C-reactive protein [11], and AFP levels [29] and some subsets of patients have been identified as having better prognosis [24, 25, 30, 31]. The causes of increased death in HCC patients with PVT include worsened residual liver function and the presence of tumor cells in the vein as a pathway to systemic spread of tumor and distant metastasis.

The mechanisms involved in the pathogenesis of PVT include disruption of the tumor matrix, migration and invasion of the tumor cells, systemic inflammation [32], and DCP [16–19]. AFP levels are typically elevated in HCC patients with PVT, but whether they are cause or consequence is unclear.

Treatment of HCC patients with PVT is controversial [5] with more than usual complications and some limited survival benefit [33, 34]. Recently, radioembolization has emerged as a safer therapy [35, 36], but its effects on survival have yet to be proved. However, some evidence suggests that chemoembolization may also be useful in the presence of PVT [37, 38]. Furthermore, Sorafenib has been found to be a safe treatment in this setting [39] and may be as effective as radioembolization. In additional, multiple different radiation modalities have been evaluated in the presence of PVT, with few differences between them [40].

A major finding in this analysis was the increase in % of patients with PVT as MTD increased (Tables 1, 2, and 3, Figure 1). We considered 2 possible explanations for this. One is that the same factors that induce tumor growth, such as stem cells or growth factors, also enhance tumor invasion and thus MTD. Alternatively, there might be some change in HCC biology beyond a certain size that is associated with more aggressive features. Table 1 shows that the proportion of very large MTD tumors with and without PVT is similar. Furthermore, within this category, average MTD is almost identical. This suggests that it is not tumor size per se. Perhaps there are 2 different growth pathways, one associated with PVT and the other not. This could explain why large tumors are well represented in the PVT positive and negative groups (Table 1). The same Table 1 also shows that both tumor multifocality and average AFP values are always significantly higher in every MTD category that has PVT patients compared to each PVT negative category, suggesting that the tumors are more aggressive in the PVT patients, whether they are larger or not. However, even in the highest AFP groups, less than 50% of the patients were PVT positive, as shown in Figure 2. Other reports have found elevated bilirubin levels in PVT patients, but our data do not really show this. Thus, the patients with PVT in this cohort have aggressive HCCs which did not destroy sufficient liver parenchyma to cause liver failure. PVT is generally classified as macroscopic or microscopic [5]. The current study was based on clinical/nonsurgical evidence (macroscopic) for PVT. Thus, a proportion of our patients classed as PVT negative macroscopically could still be PVT positive microscopically. It has also been reported that PVT seems to occur at a very early stage of HCC evolution [41]. These findings support the idea of 2 possible HCC developmental pathways, namely, HCCs with and without macroscopic PVT. Factors other than MTD and AFP must also be involved in the development of PVT.

## Figures and Tables

**Table 1:** Comparisons amongst HCC patients between portal vein thrombosis groups, in single MTD categories.

	MTD<5.0 (cm)			5.0≤MTD≤10.0 (cm)			MTD>10.0 (cm)		
Parameter*∗*	PVT(No)	PVT(Yes)	p^*ψ*^	PVT(No)	PVT(Yes)	p^*ψ*^	PVT(No)	PVT(Yes)	p^*ψ*^
	(n=701)	(n=112) (13.7%)		(n=388)	(n=199) (33.9%)		(n=84)	(n=109) (56.4%)	
									
Sex (M) (%)	145 (79.32)	88 (78.57)	0.86^∧^	319 (82.22)	164 (82.41)	0.95^ ∧^	72 (85.71)	96 (88.07)	0.63^ ∧^
Age (yr)	61.55±11.04	61.59±11.53	0.98	63.86±10.95	61.24±11.88	0.005	63.89±12.52	60.69±13.68	0.08
Cigarettes smoke (%)	159 (44.04)	32 (42.11)	0.76^ ∧^	129 (60.85)	79 (56.03)	0.37^ ∧^	26 (55.32)	32 (57.14)	0.85^ ∧^
Alcohol (%)	63 (17.65)	8 (11.11)	0.17^ ∧^	27 (13.85)	25 (18.12)	0.29^ ∧^	7 (20.00)	7 (12.07)	0.30^ ∧^
Child-Pugh (B&C)			0.01 ^ ∧^			<0.001^ ∧^			0.47 ^ ∧^
A	217 (39.19)	18 (22.22)		138 (44.23)	44 (26.04)		26 (35.14)	23 (26.44)	
B	212 (38.27)	41 (50.62)		118 (37.82)	70 (41.42)		28 (37.84)	39 (44.83)	
C	125 (22.56)	22 (27.16)		56 (17.95)	55 (32.54)		20 (27.03)	25 (28.74)	
Cirrhosis (%)	542 (79.47)	88 (87.13)	0.07 ^ ∧^	301 (77.98)	168 (85.71)	0.03^ ∧^	62 (73.81)	81 (77.88)	0.51 ^ ∧^
Albumin (g/dL)	3.15±0.76	2.95±0.76	0.02	3.11±0.75	2.97±0.71	0.07	3.04±0.74	3.00±0.63	0.60
GGTP (U/L)	148.07±186.14	143.42±134.30	0.41	147.47±153.36	210.61±206.48	0.0003	186.06±153.04	213.74±175.12	0.34
AST (U/L)	118.06±226.07	102.20±92.59	0.86	100.88±121.40	114.37±105.87	0.02	146.35±425.67	114.46±112.63	0.07
ALKP (U/L)	190.97±216.09	191.34±168.76	0.40	193.97±139.92	279.19±313.31	0.02	232.77±149.04	268.86±241.38	0.96
Bilirubin (mg/dL)	2.41±3.45	2.52±3.41	0.67	2.27±3.67	3.60±5.44	0.009	1.96±3.06	2.58±3.61	0.22
Platelet counts (10^3^/*μ*L)	139.16±85.58	141.80±90.29	0.92	161.86±89.36	180.04±108.38	0.10	226.94±141.19	227.79±127.74	0.63
AFP (IU/mL)	1774.84±13163.19	2722.60±8062.90	<0.0001	3504.43±18586.15	10976.9±54278.4	0.0007	9395.0±29325.7	18393.3±55766.2	0.02
MTD (cm)	2.91±1.01	3.13±0.94	0.03	7.00±1.73	7.47±1.66	0.0004	14.29±3.56	14.24±3.17	0.91
Nodules number (%)			0.002 ^ ∧^			<0.001^ ∧^			<0.001^ ∧^
Unifocal	507 (72.53)	62 (57.94)		271 (70.76)	98 (50.52)		67 (79.76)	55 (55.00)	
Multifocality (≥2)	192 (27.47)	45 (42.06)		112 (29.24)	96 (49.48)		17 (20.24)	45 (45.00)	
									

*∗* All values: means ± standard deviation as continuous; Frequencies and percentage (%) as categorical.

^*ψ*^ Wilcoxon rank-sum (Mann–Whitney) test; ^∧^ Chi-square test.

GGTP, gamma glutamyl transpeptidase; ALKP, alkaline phosphatase; AFP, alpha-fetoprotein; MTD, maximum tumor diameter; PVT, portal vein thrombosis.

**Table 2:** Logistic regression model of PVT *(No/Yes)*, on single variables **(A)**. Final multiple logistic regression model in stepwise method of PVT *(No/Yes)*, on all variables included together in the model **(B)**. All models in total cohort.

Parameter *∗*	OR	se(OR)	p-value	95% C.I.
(**A**)				
Platelet counts (10^3^/*μ*L)	1.002	0.001	0.003	1.001 to 1.004
Hemoglobin (g/dL)	0.899	0.034	0.005	0.835 to 0.969
GGTP (U/L)	1.001	0.0004	0.006	1.0003 to 1.0021
ALKP (U/L)	1.001	0.0004	0.002	1.0004 to 1.0019
Total Bilirubin (mg/dL)	1.042	0.021	0.04	1.002 to 1.084
Albumin (g/dL)	0.711	0.080	0.002	0.571 to 0.887
AFP (IU/mL)				
≤100 *[Ref. category]*	1			
>100	2.091	0.354	<0.001	1.500 to 2.913
MTD (cm)	1.178	0.027	<0.001	1.127 to 1.231
Tumor Nodule #	1.714	0.284	0.001	1.238 to 2.372
Cirrhosis (yes)	1.388	0.303	0.13	0.905 to 2.130

(**B**)				
ALKP (U/L)	1.001	0.0004	0.02	1.0001 to 1.0017
Albumin (g/dL)	0.758	0.093	0.02	0.595 to 0.965
AFP (IU/mL)				
≤100 *[Ref. category]*	1			
>100	1.632	0.297	0.007	1.143 to 2.331
MTD (cm)	1.166	0.027	<0.001	1.114 to 1.219
Tumor Nodule #	1.558	0.281	0.01	1.094 to 2.219

*∗* All variables included in the model were considered as continuous, except the AFP which was considered as categorical.

OR, odds ratio; se(OR), standard error of odds ratio; GGTP, gamma glutamyl transpeptidase; ALKP, alkaline phosphatase; AFP, alpha-fetoprotein; MTD, maximum tumor diameter; PVT, portal vein thrombosis.

**Table 3:** Logistic regression model of PVT (*No/Yes*), on single variables (**A**), all as categories. Final multiple logistic regression model in stepwise method of PVT (*No/Yes*), on all variables as categories, included together in the model (**B**). Combination of MTD (<5.0/≥5.0), focality (Unifocality/Multifocality), and AFP (≤100/>100) (**C**).

Parameter	OR	se(OR)	p-value	95% C.I.
(**A**)				

MTD (cm)				
<5.0 *[Ref. category]*	1			
≥5.0	3.97	0.53	<0.001	3.05 to 5.17

Tumor Nodule #				
Unifocality (n=1) *[Ref. category]*	1			
Multifocality (n≥2)	2.43	0.31	<0.001	1.90 to 3.11

AFP (IU/mL)				
≤100 *[Ref. category]*	1			
>100	2.55	0.32	<0.001	2.00 to 3.25

(**B**)				

MTD (cm)				
<5.0 *[Ref. category]*	1			
≥5.0	3.49	0.48	<0.001	2.66 to 4.57
Tumor Nodule #				
Unifocality (n=1) *[Ref. category]*	1			
Multifocality (n≥2)	2.22	0.29	<0.001	1.71 to 2.88
AFP (IU/mL)				
≤100 *[Ref. category]*	1			
>100	2.05	0.27	<0.001	1.58 to 2.65

(**C**)				

Combination of: MTD, Focality, and AFP				

MTD<5.0 & Unifocality & AFP≤100 *[Ref. category]*	1			
MTD<5.0 & Unifocality & AFP>100	2.80	0.82	<0.001	1.57 to 4.99
MTD<5.0 & Multifocality & AFP≤100	2.17	0.71	0.02	1.15 to 4.11
MTD<5.0 & Multifocality & AFP>100	6.93	2.31	<0.001	3.60 to 13.31
MTD≥5.0 & Unifocality & AFP≤100	4.68	1.20	<0.001	2.83 to 7.73
MTD≥5.0 & Unifocality & AFP>100	7.48	1.92	<0.001	4.52 to 12.38
MTD≥5.0 & Multifocality & AFP≤100	9.50	2.76	<0.001	5.37 to 16.80
MTD≥5.0 & Multifocality & AFP>100	17.94	4.80	<0.001	10.62 to 30.30

OR, odds ratio; se(OR), standard error of odds ratio; PVT, portal vein thrombosis; MTD, maximum tumor diameter; AFP, alpha-fetoprotein.

## Abbreviations

HCC: Hepatocellular carcinoma
PVT: Portal vein thrombosis
AFP: Alpha-fetoprotein
GGTP: Gamma glutamyl transpeptidase
ALKP: Alkaline phosphatase
AST: Aspartate aminotransferase
Alb: Albumin
MTD: Maximum tumor diameter
CRP: C-reactive protein
CT: Computerized axial tomography
MRI: Magnetic resonance imaging.
